# Errata

**Published:** 2011-08-01

Vrijheid et al. [Environ Health Perspect 119:598–606 (2011)] have reported errors in their paper, which resulted from errors in formulas applied to convert risk estimates for continuous exposure from reviewed studies. These errors affect the summary values in their Abstract, Figure 1, Tables 2 and 3, and Supplemental Material, Table 2A–E.

Data for Dadvand (2011a), Dolk et al. (2010), Strickland et al. (2009) and Marshall et al. (2010) have been corrected in Supplemental Material, Table 2A–E (pp. 5–14; doi:10.1289/ehp.1002946). Because several summary values were incorrect in Tables 2 and 3, the corrected tables are presented here. In addition, in Figure 1C (PM10 and ASDs), “Dolk et al. (2009)” should be “Hansen et al. (2009).” Figure 1 has been corrected online.

These errors also affect the meta-analysis estimates in the abstract (under “Data synthesis”), which have been corrected here:

In meta-analyses, nitrogen dioxide (NO2) and sulfur dioxide (SO2) exposures were related to increases in risk of coarctation of the aorta [odds ratio (OR) per 10 ppb NO2 = 1.20; 95% confidence interval (CI), 1.00–144; OR per 1 ppb SO2 = 1.04; 95% CI, 1.00–1.08] and tetralogy of Fallot (OR per 10 ppb NO2 = 1.25; 95% CI, 1.02–1.51; OR per 1 ppb SO2 = 1.04; 95% CI, 1.00–1.08), and PM10 (particulate matter ≤ 10 µm) exposure was related to an increased risk of atrial septal defects (OR per 10 μg/m3 = 1.14; 95% CI, 1.01–1.28).

None of these corrections affect the statistical significance of the pooled estimates or the conclusions of the paper.

The authors apologize for the errors.

**Table 2. Summary of meta-analysis of studies on air pollutant exposures and cardiac anomalies.** **a**Studies included are different in each pollutant-anomaly meta-analysis depending on the data they published. References are as follows: 1, Ritz et al. (2002); 2, Gilboa et al. (2005); 4, Rankin et al. (2009); 5, Strickland et al. (2009); 6, Hansen et al. (2009); 7, Dolk et al. (2010); 9, Dadvand et al. (2011b); 10, Dadvand et al. (2011a). **b**Number of cases included in the continuous exposure analysis. **c**Conversion factors: CO, 1 ppb = 1.15 μg/m^3^; NO_2_, 1 ppb = 1.88 μg/m^3^; O_3_, 1 ppb = 1.96 μg/m^3^; SO_2_, 1 ppb = 2.62 μg/m^3^. **d**When heterogeneity *p*-value is < 0.10, the OR from random effect model is shown; otherwise, the OR from the fixed-effects model is shown. **e**Sensitivity analysis using Dadvand 2011a instead of Dadvand 2011b. **f**Sensitivity analysis including Rankin 2009 instead of Dadvand 2011b.

Pollutant and anomaly combination	Total number of cases*b*	Continuous exposure*c*		High versus low exposure	
		Studies included*a*	Heterogeneity *p*-value	Summary OR (95% CI)*d*	Heterogeneity *p*-value	Summary OR (95% CI)*d*
CO						per 1 ppm			
ASDs		1, 2, 5, 6, 9	1,337		0.10	0.87 (0.72–1.05)		0.17	0.86 (0.75–0.99)
VSDs		1, 2, 5, 6, 9	3,710		< 0.001	1.14 (0.66–1.98)		< 0.001	1.18 (0.82–1.69)
Conotruncal defects		1, 2, 5, 6	1,156		0.02	0.95 (0.57–1.58)		0.01	0.95 (0.62–1.44)
NO_2_						per 10 ppb			
ASDs		2, 5, 6, 9	952		0.81	1.10 (0.91–1.33)		0.28	1.07 (0.90–1.26)
VSDs		2, 5, 6, 9	3,460		0.002	1.12 (0.87–1.44)		0.03	0.92 (0.77–1.12)
Coarctation of the aorta		2, 5, 7, 9	756		0.31	1.20 (1.00–1.44)		0.13	1.04 (0.86–1.26)
Tetralogy of Fallot		2, 5, 7, 9	704		0.23	1.25 (1.02–1.51)		0.06	1.04 (0.70–1.55)
O_3_						per 10 ppb			
ASDs		1, 2, 5, 6, 9	1,307		0.08	1.10 (0.92–1.32)		0.31	0.99 (0.83–1.19)
VSDs		1, 2, 5, 6, 9	3,557		0.04	0.94 (0.82–1.08)		0.02	0.93 (0.73–1.18)
Conotruncal defects		1, 2, 5, 6	1,164		0.64	1.07 (0.96–1.19)		0.45	1.13 (0.89–1.42)
PM_10_						per 10 μg/m^3^			
ASDs		2, 5, 6, 9	951		0.10	1.14 (1.01–1.28)		0.02	1.23 (0.91–1.67)
VSDs		2, 5, 6, 9	3,410		0.12	1.03 (0.96–1.10)		0.70	0.93 (0.84–1.02)
Coarctation of the aorta		2, 5, 7, 9	761		0.02	1.10 (0.88–1.39)		0.48	1.00 (0.79–1.26)
Tetralogy of Fallot		2, 5, 7, 9	546		0.37	1.00 (0.87–1.15)		0.02	0.91 (0.53–1.56)
SO_2_						per 1 ppb			
ASDs		2, 5, 6, 9	909		0.01	0.96 (0.86–1.07)		0.01	0.85 (0.57–1.28)
		2, 5, 6, 10*e*	914		0.01	0.97 (0.87–1.08)		0.02	0.92 (0.63–1.33)
VSDs		2, 5, 6, 9	3,217		< 0.001	1.03 (0.97–1.11)		< 0.001	0.96 (0.63–1.46)
		2, 5, 6, 10*e*	3,056		< 0.001	1.01 (0.94–1.07)		< 0.001	1.08 (0.81–1.44)
		2, 4, 5, 6*f*						< 0.001	1.05 (0.76–1.46)
Coarctation of the aorta		2, 5, 7, 9	682		0.71	1.04 (1.00–1.08)		0.29	1.06 (0.89–1.27)
		2, 5, 7, 10*e*	687		0.02	1.01 (0.94–1.07)		0.01	0.89 (0.61–1.32)
		2, 4, 5, 7*f*						0.95	1.10 (0.92–1.31)
Tetralogy of Fallot		2, 5, 7, 9	655		0.10	1.04 (1.00–1.08)		< 0.001	0.80 (0.45–1.41)
		2, 5, 7, 10*e*	670		0.05	1.03 (0.97–1.09)		0.06	1.02 (0.75–1.39)
		2, 4, 5, 7						0.13	1.13 (0.93–1.36)

**Table 3. Summary of meta-analysis of studies on air pollutant exposures and orofacial clefts.** **a**References are as follows: 1, Ritz et al. (2002); 2, Gilboa et al. (2005); 3, Hwang and Jaakkola (2008); 4, Rankin et al. (2009); 6, Hansen et al. (2009); 7, Dolk et al. (2010); 8, Marshall et al. (2010). **b**Number of cases included in the continuous exposure analysis. **c**Conversion factors: CO, 1 ppb = 1.15 μg/m^3^; NO_2_, 1 ppb = 1.88 μg/m^3^; O_3_, 1 ppb = 1.96 μg/m^3^; SO_2_, 1 ppb = 2.62 μg/m^3^. **d**When heterogeneity *p*-value is < 0.10 the OR from random effect model is shown; otherwise, the OR from the fixed-effect model is shown. **e**Cleft lip with or without cleft palate. **f**Sensitivity analysis including Rankin 2009 (no continuous exposure estimate available).

Pollutant and anomaly combination	Total number of cases*b*	Continuous exposure*c*			High versus low exposure		
		Studies included*a*	Heterogeneity *p*-value	Summary OR (95% CI)*d*	Heterogeneity *p*-value	Summary OR (95% CI)*d*
CO								per 1 ppm				
Cleft lip/palate*e*		1, 2, 3, 6, 8		1,498		0.03		0.89 (0.66–1.20)		0.002		0.99 (0.79–1.24)
Cleft palate		1, 2, 6, 8		697		0.009		0.68 (0.36–1.25)		0.06		0.78 (0.55–1.12)
NO_2_								per 10 ppb				
Cleft lip/palate		2, 6, 7, 8		1,423		0.43		1.00 (0.87–1.16)		0.25		1.06 (0.88–1.28)
Cleft palate		2, 6, 7, 8		809		0.12		0.91 (0.76–1.08)		0.23		0.79 (0.62–1.01)
O_3_								per 10 ppb				
Cleft lip/palate		1, 2, 3, 6, 8		1,950		0.20		1.10 (0.99–1.21)		0.22		1.06 (0.96–1.17)
Cleft palate		1, 2, 6, 8		702		0.79		0.99 (0.83–1.18)		0.42		1.00 (0.79–1.26)
PM_10_								per 10 μg/m^3^				
Cleft lip/palate		2, 3, 6, 7, 8		2,072		0.70		1.01 (0.96–1.06)		0.45		1.02 (0.94–1.11)
Cleft palate		2, 6, 7, 8		803		0.008		0.91 (0.58–1.41)		0.15		0.86 (0.70–1.07)
SO_2_								per 1 ppb				
Cleft lip/palate		2, 3, 6, 7, 8		1,976		0.06		0.99 (0.94–1.05)		0.01		1.06 (0.87–1.29)
		2, 3, 4, 6, 7, 8*f*								0.02		1.06 (0.89–1.27)
Cleft palate		2, 6, 7, 8		764		0.35		1.03 (0.98–1.07)		0.17		0.94 (0.81–1.10)

